# Catch-up growth in stunted children: Definitions and predictors

**DOI:** 10.1371/journal.pone.0189135

**Published:** 2017-12-13

**Authors:** Chris Desmond, Daniela Casale

**Affiliations:** 1 Developmental Pathways to Health Research Unit, University of Witwatersrand, Johannesburg, South Africa; 2 School of Economic and Business Sciences, University of Witwatersrand, Johannesburg, South Africa; CUNY, UNITED STATES

## Abstract

This paper examines the incidence and correlates of linear growth catch up in early childhood among stunted children, using a range of definitions of catch up. Catch-up growth between two and five years of age is defined in both absolute terms (i.e. the centimetre height deficit from the healthy reference population mean is reduced) and relative terms (the height-for-age z-score improved or passed the -2SD or -1SD cut-off points). Data from a cohort study from urban South Africa are used to estimate the percentage of children who caught up and the predictors of catch-up growth according to these varying definitions. The results show that our sample of stunted children exhibits catch-up growth regardless of the definition used, however prevalence of catch up is highly sensitive to the way catch up is classified, ranging from 19%-93%. Of the biological, early growth, socioeconomic status and maternal reproductive variables included in the multivariate probit regressions, only a few were found to be consistent predictors of the incidence of catch-up growth. Mother’s height was positively correlated with the incidence of catch-up growth and early stunting at one year was associated with a lower likelihood of subsequent catch up.

## Introduction

The first 1,000 days of life (pregnancy and the first two years) are critical for brain development [1]. The energy intensive nature of brain development during this period makes it sensitive to nutritional intake [2]. Malnutrition, indicated by stunting, during this critical developmental stage has been linked to a range of negative developmental outcomes, including a detrimental impact on cognitive development [3,4]. Utilizing data from cohort studies, early-life stunting (typically measured at two years of age), via its impact on development, has been shown to affect later-life outcomes, including educational attainment and adult earnings [5].

The importance of linear growth within the first 1,000 days is well-established. Debate, however, continues regarding the extent to which stunted children can catch up that growth after two years of age [6–10], and the extent to which such catch up mitigates the cognitive impacts of the early growth faltering i.e. stunting by age two [11–18]. In this paper we seek to contribute to the first part of this debate and focus on the incidence of catch-up growth among stunted children in a high risk context. Moreover, we aim to contribute to an under-researched topic in this area [19]—the factors associated with the incidence of catch up. Estimating the predictors of catch-up growth can help us to identify which children are more likely to catch up and which are at risk of staying behind or falling further behind. With the data available, however, we cannot identify causation. Nonetheless, the results may be suggestive of avenues worth exploring in subsequent causal analysis.

In this study we focus on the incidence of catch-up growth between 2 and 5 years among children stunted at 2 years. We analyse children stunted at 2 years because this population has been linked to negative developmental outcomes, and as a result catch-up growth may be critically important. Further, we analyse catch-up growth in the period immediately after the first 1,000 days, given that rapid development continues during this period, and therefore it may also be critical for later outcomes.

Catch-up growth has been defined in a number of different ways in the literature and the debate on the extent to which catch up occurs centres on the choice of which definition to use [7,9,10,14]. These can be summarised into absolute and relative definitions of catch-up growth. Both types of definition compare the child’s height to the WHO reference population benchmark and in this sense are both relative. However, the former expresses the difference in centimetres (cm), an absolute measure, while the latter measures growth in standard deviations of the reference distribution, a relative measure. It is this difference which has motivated the absolute and relative labels now common in the literature. Further detail on the definitions used is provided below.

Absolute catch-up growth can be defined as a decrease over time in the absolute height deficit, measured in cm, between the individual or population and the reference mean for a healthy population [9,10,19]. In other words, catch-up growth would occur if the cm height-for-age difference (HAD) declined, to use the nomenclature in Leroy et al [10].

Relative definitions of catch up are more commonly used in the recent literature on catch-up growth in height [13–15,18], and account for the increasing variability in height as children age, and the associated decreasing importance of a given absolute cm deficit. Given that the same absolute difference in height is less noteworthy the older the child, relative definitions assess the deficit at any age relative to the reference population distribution of height at that age. To do so, they define catch-up growth in relation to changes in the height-for-age z-score (HAZ).

HAZ is designed to identify the significance of height variations from the healthy population mean, given the expected variation about that mean. HAZ is calculated by dividing the absolute height difference from the age- and sex-specific reference mean by the standard deviation (SD) for that mean. The reference mean and associated SD increase with age. As a result, a given absolute deviation from the mean is considered less and less significant the older the population.

Relative catch-up growth can be defined simply as an improvement in the HAZ. An improvement in HAZ suggests that a child’s height is less of an outlier than it was when they were younger. This may or may not mean that their height caught up in absolute cm terms, however. If the absolute deficit remains the same, while the SD increases with age, the HAZ will decline. It is even possible that the absolute cm deficit widens and the HAZ improves, provided it widens more slowly than the SD increases. It is worth noting that changes in HAZ and HAD between any two ages, by construction, are perfectly correlated, but the definitions of catch up are different. The HAZ calculation effectively rescales the distribution, pushing a higher proportion over the zero mean of the Z-score. As a result, definitions of catch up based on positive changes in HAD or positive changes in HAZ produce different rates of catch up among children.

Another common relative definition of catch up used in the literature, albeit a stricter one, is recovery from stunting [11,12,19–21]. A child who is more than two standard deviations below the mean of the healthy reference population (i.e. is stunted) at a given age, is classified as having recovered from stunting if he/she has an improvement in HAZ such that at some later age the child’s HAZ is above the -2 SD cut-off.

Analyses based on relative definitions of catch up, including recovery from stunting, have been criticised [9,10]. That a child can fall further behind the reference mean in absolute terms yet still be defined as having exhibited catch-up growth is seen as problematic. The recovery from stunting definition also suffers from the problem that children close to the cut-off line to start with are more likely to be classified as having exhibited catch-up growth than those initially further away. Absolute definitions of catch up are stricter and more intuitive. They do, however, ignore the increasing variability in height as children age, and what this implies for the noteworthiness of a given absolute deficit. Relative measures may, therefore, still be instructive.

We use data from the South African Birth to Twenty Plus cohort to examine the sensitivity of estimates of the incidence of catch up to the definition of catch-up growth applied. South African data is useful for this purpose as there are high rates of growth faltering in the first 2 years, then there is documented catch up in relative terms by 5 years [22]. We examine if there is only relative catch up or also absolute catch up. For each definition, we then examine which factors are associated with the incidence of catch-up growth. The comprehensive nature of the data set allows us to examine the association with a range of biological, early growth, socio-economic and maternal reproductive variables.

## Data and methods

Birth to Twenty Plus is a cohort study of children born in a seven-week period between April and June 1990 in Johannesburg, South Africa’s largest metropole. In addition to a wide array of child, maternal and household characteristics collected through face-to-face interviews with the caregiver and child, anthropometric data were measured regularly in the early childhood period. There were 3273 eligible singleton births over the study period, however for the years which we are interested in for our analysis, namely when the children were aged two and five years, the number of children interviewed was 1839 and 1586 respectively. The characteristics of the cohort and attrition over the course of the study have been described extensively elsewhere. Loss to follow-up was due to two main reasons: the mothers of white, middle-class children, living in the more affluent suburbs, had higher rates of non-response; and a substantial number of African mothers and their children were lost within the first year of the study, as it is likely that they came to the city to give birth in better public health facilities and then returned to rural areas thereafter [23–25]. Nonetheless, there are no significant differences between the original sample of children and the sample of children who appear at both 2y and 5y and have data on height (n = 1578) on other key variables relevant to our analysis, such as the child’s gender and birth weight and the mother’s height.

### Definitions of catch-up growth

To investigate the incidence and correlates of catch-up growth in our sample, we explore both absolute and relative definitions. As explained in the introduction, absolute catch up would require that the child grow sufficiently to start closing the absolute (cm) height deficit in relation to the mean height of the healthy reference population (we use the WHO 2006 standards). In other words, using the terminology in Leroy et al [10] the height-for-age difference (HAD) would need to narrow over the period of analysis, HAD calculated as the child’s observed height minus the age and sex-specific median reference height (in cm). A relative definition of catch up would require only that the HAZ improved over the period of analysis, HAZ calculated as the HAD/SD, where SD is the age- and sex-specific standard deviation.

Table 1 provides summary statistics on linear growth for our sample. These are shown for all children who had a valid height measurement at 2 years and 5 years (n = 1576), and for the sub-samples of children who were not stunted at 2 years (n = 1272) and those that were stunted at 2 years (n = 304). Based on this, the prevalence of stunting at age two was 19.3% (304/1576).

**Table 1:** Summary statistics on growth.

	ALL CHILDREN (n = 1576)				NON-STUNTED (n = 1272)				STUNTED AT 2 YEARS (n = 304)			
Variable	Mean	SD	Min	Max	Mean	SD	Min	Max	Mean	SD	Min	Max
**YEAR 2**												
Observed height (cm)	83.40	3.40	70.00	97.00	84.49	2.64	79.29	97.00	78.82	2.15	70.00	83.00
Reference height (cm)	86.95	1.15	85.04	91.07	86.85	1.11	85.10	91.07	87.37	1.20	85.04	90.76
Height-for-age difference (HAD) (cm)	-3.56	3.40	-17.50	9.35	-2.36	2.42	-6.80	9.35	-8.55	2.06	-17.50	-5.99
Height-for-age z-score (HAZ = HAD/SD)	-1.13	1.07	-5.68	2.91	-0.75	0.76	-2.00	2.91	-2.70	0.65	-5.68	-2.01
**YEAR 5**												
Observed height (cm)	107.26	4.47	94.00	124.60	108.29	4.04	97.6	124.60	102.96	3.50	94.00	114.30
Reference height (cm)	110.37	1.00	106.95	113.43	110.36	0.99	107.77	113.43	110.39	1.04	106.95	113.43
Height-for-age difference (HAD) (cm)	-3.11	4.23	-16.28	13.95	-2.08	3.76	-13.46	13.95	-7.44	3.23	-16.28	2.90
Height-for-age z-score (HAZ = HAD/SD)	-0.66	0.90	-3.49	2.94	-0.44	0.79	-2.78	2.94	-1.58	0.69	-3.49	0.66

The sample is restricted to those children who had valid height values at 2 years and 5 years. Children who had a HAZ of less than -6 were removed (n = 2).

For the full sample of children, the mean height-for age difference was -3.56cm at two years and by five years this had reduced to -3.11cm, indicating population-level absolute catch-up growth compared to the mean reference height. Catch-up growth in relative terms, reflected in an improvement in HAZ, was more pronounced as one would expect given its construction, with a mean HAZ at two years of -1.13 and at 5 years of -0.66. Children not stunted at 2 years were on average still below the reference mean and both the HAD and HAZ deficits fell between 2 and 5 years, although not markedly. For the sample of children who had already fallen behind by two years, i.e. who were stunted at 2 years, both absolute and relative catch-up growth was also evident: mean HAD was -8.55cm at 2 years and improved to -7.44cm at 5 years and mean HAZ for this group increased from -2.70 at 2 years to -1.58 at 5 years.

While Table 1 shows mean or population-level catch-up growth, in this paper we are particularly interested in the incidence of individual-level catch-up growth among children who were stunted at two years. Stated differently, among those stunted at two years, we calculate the percentage of children who experienced catch-up growth by 5 years and we estimate the predictors of individual catch up. We use five definitions to identify the incidence of catch-up growth for our analysis, ranging here from the weakest to the strictest definition: a) the child experienced an increase in HAZ between 2 and 5 years (a simple relative definition); b) the child experienced an increase in HAZ, such that by 5 years HAZ >-2, i.e. the child had recovered from stunting (a relative definition with a cut-off); c) the child experienced an increase in HAD between 2 and 5 years (a simple absolute definition); d) the child experienced a recovery from stunting (HAZ>-2 at 5 years) *and* an increase in HAD (recovery from stunting and absolute catch-up); and e) the child experienced a recovery from stunting such that HAZ at 5 years fell within the ‘normal’ range of >-1 (recovery from stunting with a stricter cut-off point) [26]. Under a normal distribution, 15.87% of the population would fall below the -1 HAZ cut-off, or in other words we define as ‘normal’ being at around the 16th percentile and above, a definition which has previously been applied [26].

### Multivariate analysis

To investigate the predictors of the incidence of catch-up growth, we estimate probit regressions for the sample of children who were stunted at two years, using the five definitions of the incidence of catch-up growth described above as the binary outcome variables.

We include four sets of variables, based on our reading of the relevant literature on the predictors of catch-up growth [12,13,19,20]. The first set of ‘biological’ characteristics includes the child’s gender and the mother’s height, the latter capturing to some extent the child’s inherited potential for growth. The second set of ‘early growth’ variables controls for the child’s birthweight and whether the child was already stunted by one year. We would predict that children who have fallen behind early on, would find it harder to catch up subsequently. The third set includes ‘socioeconomic status’ variables, namely mother’s schooling (in years) and a household asset index from when the child was two years old (derived from principal components analysis of six assets available in the survey—fridge, car, washing machine, television, phone and radio). Socioeconomic status is predicted to be positively correlated with the incidence of catch-up growth. The fourth set of ‘maternal reproductive behaviour’ variables contains mother’s age at the child’s birth, birth order, and birth spacing (a dummy variable equal to one if another child was born within 24 months of the index child). Mothers who are older are likely to have greater reproductive autonomy–also signalled by a lower number of children and a larger spacing between children [27] -which we would predict to be positively correlated with the incidence of catch-up growth. Fewer children and wider birth spacing would place less strain on limited financial resources and on the mother’s attention and energy, improving the index child’s chances of recovery.

Table 2 presents the summary statistics of the variables used in the multivariate analysis for the sample of children stunted at 2 years. Just under 45% of the sample is female. Average maternal height is 156.5cm, mean birthweight 2866 grams, and 33% of the sample of children stunted at two years was already stunted by one year. Mothers had 9.3 years of schooling on average and households had a mean of 3.5 out of 6 potential assets. Mean maternal age at birth was 25 years, the mean index child was second-born, and only 8% of mothers had another child within 24 months of the index child.

**Table 2:** Summary statistics of variables in regression analysis.

	N	Mean	SD	Min	Max
**Biological factors**					
Female	304	0.44	0.50	0	1
Mother’s height (cms)	232	156.5	5.54	143	170
**Growth 0-1y**					
Birth weight (grams)	304	2866	539	1070	4800
Stunted at 1y	297	0.33	0.47	0	1
**SES**					
Mother’s education (years)	302	9.28	2.82	0	14
Asset index (y2)	290	3.48	1.47	0	6
**Maternal reproductive behaviour**					
Mother’s age at child’s birth	304	25.16	6.53	15	46
Birth order	304	2.13	1.13	1	4
Birth spacing^a^	288	0.08	0.27	0	1

The sample is restricted to those children who had valid height values at 2 years and 5 years, and were stunted at 2 years. Children who had a HAZ of less than -6 were removed (n = 2).

^a^Birth spacing is an indicator variable for whether another child was born within 24 months of the index child.

We tested various additional predictors in alternative regressions, included a dietary diversity index at one year, the length of breastfeeding, symptoms of infection (respiratory, gastrointestinal, and ears and eyes, collected only at 6 months, 1 year and 2 years), whether the mother was the main caregiver, and the interviewer’s perception of the mother and child’s general well-being. None of these variables was significant in any of the regressions and substantially reduced our sample size, so we reverted to a more parsimonious regression specification.

## Results

Table 3 shows the incidence of individual-level catch-up growth using our five definitions of catch up. Most children who were stunted at two years—93%—experienced a positive change in HAZ by age five, that is, their relative height deficit was reduced (definition a). If we add a threshold to the relative definition such that children were required to have passed the -2SD HAZ cut-off at age five, then 75% of the sample caught up or had recovered from stunting by five years (definition b). Defining catch-up growth in absolute terms, so that children were required to grow sufficiently by age five to reduce the absolute cm deficit in HAD, produces a much smaller catch-up rate of 63% (definition c). Combining definitions b and c to require that children not only recover from stunting by five years (HAZ >-2 at 5y) but that they also grow sufficiently to reduce the absolute height deficit (a positive change in HAD), then 59% of children caught up (definition d). Finally, a much stricter definition of catch up that requires children to have crossed the -1 HAZ threshold into the ‘normal’ range, indicates that only 19% of the children who were stunted at two years caught up by five years (definition e).

**Table 3:** Percentage of stunted sample at 2y that experienced catch up between 2y and 5y, using different definitions of catch up.

	Percentage	n
a)Positive change in HAZ	93.1	283
b)Recovery from stunting (HAZ at 5y >-2)	75.3	229
c)Positive change in HAD	62.5	190
d)Recovery from stunting (HAZ at 5y >-2) + positive change in HAD	58.9	179
e) Recovery from stunting (HAZ at 5y >-2) + HAZ at 5y > -1 (‘normal’ range)	18.8	57
Total n stunted at 2y		304

Sample consists of children who were stunted at 2y and had a valid HAZ at 5y. Children who had a HAZ of less than -6 were removed (n = 2). If the sample is restricted to the sample of 213 children included in the regressions below, the rates of catch up are very similar to those shown here.

The results from the probit regressions estimating the predictors of the incidence of catch-up growth are displayed in Table 4. Of the biological factors, mother’s height is significant in predicting the likelihood of catch up when definitions b, c and d are used. Having a taller mother increases the chances of experiencing catch-up growth, when catch up implies a recovery from stunting and/or an increase in HAD. However the size of the coefficient becomes smaller as the definition of catch up becomes stricter, and for the strictest definition, crossing into the normal range with a HAZ >-1, mother’s height has no association.

**Table 4:** Multivariate probit regression analysis of the incidence of catch up between 2y and 5y for the sample of children stunted at 2y, using different definitions of catch up.

	a) Positive change in HAZ	b)Recovery from stunting (HAZ at 5y >-2)	c)Positive change in HAD	d)Recovery from stunting (HAZ at 5y >-2) + positive change in HAD	e) Recovery from stunting (HAZ at 5y >-2) + HAZ at 5y > -1 (‘normal’ range)
**Biological factors**					
Female	-0.211	-0.128	0.043	-0.046	-0.262
	(0.354)	(0.214)	(0.189)	(0.188)	(0.220)
Mother’s height	0.017	0.048**	0.033**	0.031*	0.019
	(0.030)	(0.019)	(0.016)	(0.016)	(0.020)
**Growth 0-1y**					
Birth weight	-0.001	0.000*	0.000	0.000	0.000
	(0.000)	(0.000)	(0.000)	(0.000)	(0.000)
Stunted at 1y	-0.916**	-0.707***	-0.528***	-0.653***	-1.383***
	(0.392)	(0.223)	(0.201)	(0.202)	(0.349)
**SES**					
Mother’s education	-0.026	0.117***	0.028	0.070*	0.018
	(0.078)	(0.044)	(0.039)	(0.039)	(0.046)
Asset index	0.165	-0.004	-0.038	-0.044	-0.013
	(0.125)	(0.078)	(0.069)	(0.069)	(0.078)
**Maternal reproductive behaviour**					
Mother’s age at child’s birth	-0.056	-0.009	-0.023	-0.025	0.000
	(0.039)	(0.027)	(0.024)	(0.024)	(0.028)
Birth order	0.155	0.112	0.098	0.166	0.149
	(0.232)	(0.157)	(0.141)	(0.141)	(0.172)
Birth spacing	-0.370	-0.533	-0.369	-0.316	-0.179
	(0.545)	(0.372)	(0.344)	(0.347)	(0.434)
Constant	1.900	-8.611***	-4.667*	-4.957*	-4.223
	(4.798)	(3.124)	(2.638)	(2.644)	(3.164)
N	213	213	213	213	213

The sample is restricted to those children who had valid height values at 2 years and 5 years, and were stunted at 2 years. Children who had a HAZ of less than -6 were removed (n = 2).

* p< = 0.1

** p< = 0.05

*** p< = 0.01

Birth weight has a positive association with the likelihood of catch up only for definition b, however whether the child was already stunted at one year greatly reduces the chances of the child subsequently exhibiting catch-up growth in all five regressions. This suggests that children whose postnatal environment results in such substantial growth retardation that they were already behind the reference population by 2SD at one year, are unlikely to be able to grow fast enough to catch up by five years, regardless of the definition used. This group might also include some children who are simply small, and would be small regardless of their environment, and therefore would not be expected to catch up.

Of the SES factors, only mother’s education had a positive association with the chances of catch up and only in two cases—when catch up was defined as a recovery from stunting (definition b), and as a recovery from stunting in conjunction with a positive change in HAD (definition d). The asset index had no association with the incidence of catch up in any of the regressions. We also tried to capture changing circumstances in the household by including a variable representing the change in the household asset count between age two and four years, but no association was found between this variable and the incidence of catch up and so we excluded it from the final regression to maximise our sample size. The lack of significance might be because there was not much change in the household’s wealth—only 26% of the regression sample experienced an increase in the asset count between two and four years, and of these, the vast majority (83%) only saw the asset index increase by 1 (in other words, the household had acquired only one additional asset in two years). None of the maternal reproductive variables had a significant association with catch up.

It is possible that some of the variables are not strongly associated with the incidence of catch-up growth because they act through other predictors included in the analysis. For instance, the effect of mother’s height is likely to be mediated by both birthweight and stunted at 1 year, so by including all three variables in the regressions we are only able to identify the influence of mother’s height on the incidence of catch up that operates over and above the association realised through the child’s growth before 2 years. Indeed, when we exclude the variables birthweight and stunted at 1y from the regressions, the size of the association between mother’s height and the incidence of catch up increases in all the regressions, but it remains insignificant in the first regression and is only marginally significant in the final regression at the 10% level. The results on the other variables also do not change substantively.

The same issue exists for the association between birthweight and catch up, when controlling for stunting at age 1. When we exclude only stunted at age 1, the association between birthweight and the incidence of catch up strengthens marginally for the final regression only (the coefficient becomes significant at the 10% level), while the other results remain largely the same.

## Discussion

The cohort of children in the Birth to Twenty Plus study experienced high levels of early-life stunting, 19% (304/1,576) at 2 years of age. The incidence of catch up by 5 years among the group of stunted children is highly sensitive to the definition of catch up that is used. If the weakest definition of catch up is applied, i.e. a positive change in HAZ between two and five years, 93% of children can be said to have exhibited catch-up growth. If the strictest definition of catch up is applied, i.e. recovery from stunting to within 1 SD of the mean by five years, 19% of the children can be said to have exhibited catch-up growth.

Although estimates of the incidence of catch up are sensitive to definition, evidence of catch-up growth does not disappear, even when the stricter definitions are applied. A large proportion of children (63%) caught up in absolute terms (i.e. the cm height deficit was reduced), and the majority of these children no longer met the definition of stunted by five years of age.

Similar patterns of stunting and recovery have been observed in other South African data sets. Figures from the cross-sectional 2012 National Health and Nutrition Examination Survey [28] suggest prevalence rates of stunting among 0–3 year olds of 26% for girls and 27% for boys, but among 4–6 year olds prevalence was only 10% for girls and 14% for boys. Similarly, estimates based on the National Income Dynamics Survey panel suggest that of the children 6 months– 2 years who were stunted in wave 1 (2008), 80% had recovered from stunting by Wave 4 (2012) when they were aged 4–6 years (own calculations).

Relative catch-up growth in early childhood has been observed in a number of contexts; examples include recovery from stunting in Peru [12], increased HAZ in Ethiopia [8], and increased HAZ in 54 resource-poor countries [14]. However, Leroy et al [10] argue that these examples of catch-up growth may merely be an artefact of the definition used. As discussed in the introduction, HAZ improves even if HAD remains constant. In their analysis of six Demographic and Health Surveys, they use the absolute definition of catch up and found no population-level catch-up growth. Our analysis focuses on frequency of individual catch up among stunted children and not on whether the average deficit from the norm declines. However, in our descriptive tables, we showed that both the average HAZ and HAD declined. Therefore, unlike Leroy et al [10], we find that in the South African setting, catch up occurs regardless of the definition applied.

Our analysis of the correlates of the incidence of catch up found that the weakest and the strictest definitions of catch-up growth were the hardest to predict. For the weakest definition, an increase in HAZ, almost all children (93%) caught up. As a result this definition provides a blunt and rather uninformative instrument with which to measure catch up. Moreover, many of the positive HAZ changes may be attributable to regression to the mean [10]. A larger proportion of the variation in catch up is explained when the definition of catch up requires children to have passed a threshold, i.e. recovered from stunting by five years of age and/or reduced the absolute height deficit. The inclusion of a threshold, or at least the requirement that there be an absolute reduction in height, may help to exclude those HAZ improvements which were only regression to the mean. The variables included in the model were also less useful in predicting the incidence of catch up according to the strictest definition, which requires children to have crossed over into the normal HAZ range of >-1. This may be because the variables included were not sufficient. If better data were available on changing feeding practices, periods of illness and medical intervention over time, for example, we may have been better able to predict this very fast catch up. Alternatively it may be that there is something particular to this group, such that their growth pattern is different (although they were behind at age two, they may have been slow starters).

We note other limitations of the analysis that may have affected results. First, as mentioned above, there were high levels of attrition in the early years of the cohort; mainly white mothers and rural women returning home. The former are unlikely to have experienced high rates of stunting, leaving little opportunity for catch up. The latter may well have had high rates of both stunting and catch up, but we have no reason to think that the factors associated with catch up would be any different, had they stayed in the city. However, they may well have been different in the rural areas to which they returned. The results, therefore, should be considered reflective of an urban cohort. Second, any measurement error in the dependent variable is exacerbated by the use of binary outcomes. Binary outcomes were used because this allows a link to the literature on the impact of stunting, which almost always utilizes stunting incidence. When we repeated the analysis using changes in HAD and HAZ as the outcome variables, however, we found it did not make a substantial difference to the overall results, and only the negative association between being female and catch up strengthened somewhat. Third, as with all analysis of this type, there is a possibility of omitted variable bias, one of the reasons we are careful not to infer causation.

Our finding that the incidence of catch-up growth is associated with maternal height is similar to previous studies of catch-up growth among children of a similar age range [13,19,20]. However, unlike these previous studies, we did not find a consistent association with living standards or maternal education. This may in part be explained by the relatively homogenous nature of the population on which we have data. There are relatively high rates of maternal education and little variation in living standards among our urban cohort. Similarly, we did not find a relationship with birth order or spacing, again possibly because the group is relatively homogenous and birth spacing followed a common pattern. We did find a consistent negative association between being stunted at one year and the probability of subsequent catch-up growth (regardless of definition). Schott et al, [13] similarly found stunting at 1 year of age to be a strong predictor of later variation.

However, the extent to which our results are comparable with these other studies is limited by differences in the variables used and the samples analysed. Adair examined the predictors of higher than expected growth among children stunted at 2 years of age, Schott et al analysed the predictors of changes in HAZ for the entire population, not only children who were stunted, and Georgiadis et al examined the predictors of changes in HAD for the entire population [13,19]. Our result for recovery from stunting (regression b), however, can be compared more easily to Crookston et al [12]. In their analysis of data from Peru they examined the predictors of recovery from stunting. Their initial measurement was, however, at a slightly younger age (an average of 12 months compared to our 24 months). They similarly found a positive association with maternal height and a strong negative association with early stunting.

In our South African data set we find high rates of stunting by two years of age and high rates of catch up by five years. The incidence of catch up is sensitive to the definition used, but even with strict definitions, it is still observed. For the weakest and strictest definitions of catch up, we struggle to find correlates. However, for the more moderate definitions, we do find a number of associations. Although causality cannot be identified, these associations do provide a signpost to possible fruitful areas of intervention or further research. For example, both maternal height and early stunting are consistently significant in predicting the incidence of catch up. If this relationship turns out to be causal, it suggests that better maternal nutrition and very early child monitoring and intervention are key.

An interesting question for future analysis is whether different definitions of catch up imply different relationships with functional correlates. Previous work using this data set showed a strong negative relationship between stunting at 2 and cognitive outcomes at five years, even among those who had recovered from stunting by age five [21,29]. An important unanswered question is whether the finding of greater cognitive deficits is robust to different definitions of catch-up growth in height.

## Supporting information

S1 File Final analysis dataset_Desmond and Casale_anon.dta. (DTA)
